# 

**Published:** 1909-03

**Authors:** 


### THE BRISTOL

### Medico-Chirurgical Journal.

A JOURNAL OF THE MEDICAL SCIENCES FOR THE WEST OF ENGLAND AND SOUTH WALES

*PUBLISHED UNDER THE AUSPICES OF*

*THE BRISTOL MEDICO-CHIRURGICAL SOCIETY.*

EDITOR:

R. SHINGLETON SMITH, M.D., B.Sc.,

WITH WHOM ARE ASSOCIATED

J. MICHELL CLARKE, M.A., M.D., J. LACY FIRTH, M.S., M.D., AND JAMES SWAIN, M.S., M.D.

ASSISTANT-EDITOR:

P. WATSON WILLIAMS, M.D.

EDITORIAL SECRETARY:

J. A. NIXON, B.A., M.B.

"Scire est nescire, nisi id me

Scire alius sciret."

LIBRARY

SURGEON GENERAL'S OFFICE

MAY 18 1910

205398.

VOL. XXVII.

BRISTOL: J. W. ARROWSMITH.

LONDON: J. & A. CHURCHILL, 7 GREAT MARLBOROUGH STREET.

1909.

WI
BR323

J.W. Arrowsmith Publisher Bristol

### CONTENTS OF VOLUME XXVII.

*Page*

The Long Fox Lecture: Metastatic Inflammations of the Eye. By J. HERBERT PARSONS, B.S., D.Sc. Lond., F.R.C.S. .. .. 1

The Causes of Transient Cerebral Paralyses. By GEORGE PARKER, M.A., M.D. Cantab. .. .. .. .. .. .. .. 15

The Physique of Boys. By BERTRAM M. H. ROGERS, M.D., B.Ch., B.A. Oxon. .. .. .. .. .. .. .. .. 27

Thomas Dover: Physician and Merchant Adventurer. By J. A. NIXON, M.B. Cantab., M.R.C.P. .. .. .. .. .. 31

Vomiting Connected with Anæsthesia. By A. L. FLEMMING, M.R.C.S. 40

Milk from a Public Health Standpoint—City of Bristol. By D. S. DAVIES, M.D. Lond., D.P.H. .. .. .. .. .. 44

The Adulteration of Milk in Bristol. By EDWARD RUSSELL, B.Sc., F.I.C. .. .. .. .. .. .. .. .. 47

Some Impurities of Vended Milks. By I. WALKER HALL, M.D. Vict. 48

The Value of Some Lactic Acid Ferments. By I. WALKER HALL, M.D. Vict. and W. A. SMITH, M.A., M.B. Oxon. .. .. 56

Precautions to be observed in Administration of Milk in Disease. By J. MICHELL CLARKE, M.A., M.D. Cantab., F.R.C.P. .. .. 57

The Heating of Milk as it Affects the Nutrition and Health of the Infant. By J. M. FORTESCUE-BRICKDALE, M.A., M.D. Oxon. .. 61

Small-pox in Bristol and Neighbourhood (1908–9). By D. S. DAVIES, M.D.Lond. .. .. .. .. .. .. .. 98

The Indications for Gastro-Enterostomy. By T. CARWARDINE, M.S.Lond., F.R.C.S. .. .. .. .. .. .. .. 107

A Family with a Congenital Deformity of the Nose, and the Results of Subcutaneous Injection of Wax. By E. H. E. STACK, M.B., B.C. Cantab., F.R.C.S. .. .. .. .. .. .. 119

A Case of Lead Encephalopathy. By J. ANGELL JAMES, M.R.C.S., L.R.C.P. .. .. .. .. .. .. .. .. 124

Cretinism and the Puerperium: Sequel to a Recorded History. By DAVID A. ALEXANDER, M.B. .. .. .. .. .. 128

CONTENTS.

| | Page |
|---|---|
| A Discussion on "Treatment by Ionisation or Cataphoresis." Opened by H. P. Tayler, M.A., M.B.Cantab., and H. Lewis Jones, M.A., M.D.Cantab., F.R.C.P. .. .. .. .. | 132 |
| Electricity in General Practice. By Herbert P. Tayler, M.A., M.B.Cantab. .. .. .. .. .. .. .. .. | 145 |
| Referred Cardiac Pain. By F. G. Thomson, B.A.Cantab., M.D.Lond. | 193 |
| Tuberculosis of the Colon. By Ernest W. Hey Groves, M.S., B.Sc., F.R.C.S. .. .. .. .. .. .. .. .. | 203 |
| Theoretical Considerations of Pulmonary Percussion Notes. By George Hely-Hutchinson Almond, M.A., M.B.Oxon... .. | 215 |
| Some Points in the Technique of the Radical Operation for Chronic Otitis Media Purulenta. By P. Watson Williams, M.D.Lond. | 224 |
| Sleep and the Modern Hypnotics. By J. M. Fortescue-Brickdale, M.A., M.D.Oxon. .. .. .. .. .. .. .. | 230 |
| The Safety of the Patient in Anæsthesia. By John Freeman, M.D.Durh., F.R.C.S.Edin. .. .. .. .. .. .. | 238 |
| The History and Practice of Surgery in Ancient and Mediæval Times. By James Swain, M.S., M.D. Lond., F.R.C.S. .. .. .. | 289 |
| The Mercurial Treatment of Tabes Dorsalis. By Maurice Faure, M.D. .. .. .. .. .. .. .. .. .. | 312 |
| "Vis Medicatrix Naturæ." By G. Munro Smith, M.R.C.S. .. | 321 |
| Cases of Pneumonia. By Arthur Fells, M.B., C.M. Edin. .. .. | 336 |
| Progress of the Medical Sciences .. 62, 152, 240, | 339 |
| Reviews of Books .. .. .. .. 71, 165, 247, | 349 |
| Editorial Notes .. .. .. .. 78, 178, 269, | 368 |
| Notes on Preparations for the Sick .. 85, 183, 279, | 374 |
| The Library of the Bristol Medico-Chirurgical Society .. .. .. .. 88, 184, 282, | 377 |
| Meetings of Societies .. .. .. .. .. 91, 186, | 378 |
| Local Medical Notes .. .. .. .. 93, 189, 284, | 383 |

### The Library of the Bristol Medico-Chirurgical Society.

*The following donations have been received since the publication of the List in December:*

*February 27th,* 1909.

| | |
|---|---|
| L. M. Griffiths (1) .. .. .. .. .. | 93 volumes. |
| Medical Officer of the London County Council (2) | 1 volume. |
| The Pathological Society of London (3) .. .. | 1 ,, |
| The Surgeon-General, United States Army (4) .. | 1 ,, |
| J. Taylor (5) .. .. .. .. .. .. | 1 ,, |

### SEVENTY-FIRST LIST OF BOOKS.

The titles of books mentioned in previous lists are not repeated.

The figures in brackets refer to the figures after the names of the donors, and show by whom the volumes were presented. The books to which no such figures are attached have either been bought from the Library Fund, or received through the *Journal.*

**Barr, T. and J. S.** *Diseases of the Ear* .. .. .. .. 4th Ed. 1909

**Bennett, Sir W.** *Injuries and Diseases of the Knee-Joint* .. .. .. 1909

**Bland-Sutton, J.** *On the Position of Abdominal Hysterectomy in London* .. .. .. .. .. .. .. .. .. 1909

**Brunton and L. Rogers, Sir J. Fayrer, Sir L.** *On the Poison of Venomous Snakes* .. .. .. .. .. .. .. .. 1909

**Burghard, F. F.** [Ed.] *A System of Operative Surgery.* Vol. I. .. 1909

**Buzzard, I. B. Yeo, R. Crawfurd, and E. F.** *A Manual of Medical Treatment.* 2 Vols. .. .. .. .. 4th Ed. 1909

*Catalogue (Index-) of the Library of the Surgeon-General's Office, United States Army.* 2nd Ser. Vol. XIII. (4) 1908

**Cazenave et H. E. Schedel, A.** *Abrégé pratique des Maladies de la Peau* .. .. .. .. .. .. (1) 3me Éd. 1838

**Civiale [J.]** .. *Traité pratique sur les Maladies des Organes génito-urinaires.* 3 Vols. .. .. (1) 2me Éd. 1842–50

**Crawfurd and E. F. Buzzard, I. B. Yeo, R.** *A Manual of Medical Treatment.* 2 Vols. .. .. .. .. 4th Ed. 1909

**Dawson, E. R.** *The Causation of Sex* .. .. .. .. .. .. 1909

**Dupuytren [G.]** *Leçons orales de clinique chirurgicale.* 6 Vols. (1) 2me Éd. 1839

**Fayrer, Sir L. Brunton and L. Rogers, Sir J.** *On the Poison of Venomous Snakes* .. .. .. .. .. .. .. .. 1909

**Jordan, F.** .. *Surgical Enquiries* .. .. .. .. .. (1) 2nd Ed. [N.D.]

**Keen, W. W.** [Ed.] *Surgery.* Vol. I–IV. .. .. .. .. .. .. 1908

**Kipling, R.** .. *Doctors* .. .. .. .. .. .. .. .. .. .. 1908

**Lamb, W.** .. *Diseases of the Throat, Nose and Ear.* .. 2nd Ed. 1909

**Lawrence, W.** *Venereal Diseases of the Eye* .. .. .. .. .. (1) 1830

**Lisfranc, J.** .. *Clinique chirurgicale de l'Hopital de la Pitié.* 3 Vols. .. .. .. .. .. .. .. .. .. (1) 1841–43

**Marsden, R. W.** *Infectious Disease* .. .. .. .. .. .. .. 1908

**Meadows, B.** .. *Eruptions* .. .. .. .. .. .. .. (1) 7th Ed. 1875

**Meigs, A. V.** *Milk Analysis and Infant Feeding* .. .. .. .. (1) 1885

**Philip, F. R.** .. *De Clavo secalis* .. .. .. .. .. .. .. .. (1) 1826

**Ramsay, A. M.** *Diathesis and Ocular Diseases* .. .. .. .. .. 1909

**Rogers, Sir J. Fayrer, Sir L. Brunton and L.** *On the Poison of Venomous Snakes* .. .. .. .. .. .. .. .. 1909

**Schedel, A. Cazenave et H. E.** *Abrégé pratique des Maladies de la Peau* .. .. .. .. .. (1) 3me Éd. 1838

**Scoresby-Jackson, R. E.** *Materia Medica* .. .. (1) 3rd Ed. 1875

**Scott, C. E. West and S. R.** *The Operations of Aural Surgery* .. .. 1909

**Spencer, C. G.** *Gunshot Wounds* .. .. .. .. .. .. .. .. 1908

**Stoddart, W. H. B.** *Mind and its Disorders* .. .. .. .. .. 1908

**Sturges, O.** .. *Introduction to Clinical Medicine* .. .. .. (1) 1873

**Tanner, T. H.** *Memoranda on Poisons* .. .. (1) 2nd Ed. 1862

**Thorne, L. T.** *The "Nauheim" Treatment of Diseases of the Heart* .. .. .. .. .. .. .. .. 3rd Ed. 1909
**Wanklyn, J. A.** *Water Analysis* .. .. .. .. .. (1) 5th Ed. 1879
**Waring, H. J.** *Manual of Operative Surgery*.. .. .. 3rd Ed. 1909
**West and S. R. Scott, C. E.** *The Operations of Aural Surgery* .. .. 1909
**White, C. P.** .. *Lectures on the Pathology of Cancer* .. .. .. 1908
**White, F. F.** .. *Infected Ears* .. .. .. .. .. .. .. .. .. .. .. 1908
**Yeo, R. Crawfurd and E. F. Buzzard, I. B.** *A Manual of Medical Treatment.* 2 Vols. .. .. .. 4th Ed. 1909

### TRANSACTIONS, REPORTS, JOURNALS, &c.

American Journal of Obstetrics, The .. .. .. .. Vol. LVIII. 1908
American Journal of the Medical Sciences, The .. Vol. CXXXVI 1908
American Ophthalmological Society, Transactions of the Vol. XI, Pt. 3 1908
American Proctologic Society, Transactions of the .. .. .. .. 1908
American Surgical Association, Transactions of the Vol. XXVI 1908
Association of American Physicians, Transactions of the Vol. XXVIII 1908
Bristol Directory, Wright's .. .. .. .. .. .. .. .. .. .. .. .. .. 1909
Bristol Health Report for 1907 .. .. .. .. .. .. .. .. .. .. 1908
British Medical Journal, The .. .. .. .. .. .. .. .. Vol. II for 1908
Edinburgh Medical Journal .. .. .. .. .. .. .. .. N.S., Vol. I 1908
Gazette hebdomadaire des Sciences médicales de Bordeaux Tome XXIX 1908
Hazell's Annual .. .. .. .. .. .. .. .. .. .. .. .. .. .. .. .. 1909
Hospital, The .. .. .. .. .. .. .. .. .. .. .. .. Vol. XLIV 1908
Hospitals—
Guy's Hospital Reports .. .. .. .. .. .. .. .. Vol. LXII 1908
International Congress of School Hygiene, Transactions of the Second .. .. .. .. .. .. .. .. .. .. .. .. .. 3 Vols. 1907
Journal of Laryngology, The .. .. .. .. .. .. .. Vol. XXIII 1908
Lancet, The .. .. .. .. .. .. .. .. .. .. .. .. Vol. II for 1908
London County Council, Report of the Medical Officer of Health of the, for 1907 .. .. .. .. .. .. .. .. .. .. .. .. .. (2) 1909
Medical Chronicle, The .. .. .. .. .. .. .. N.S., Vol. XV 1908
Medical Directory, The .. .. .. .. .. .. .. .. .. .. .. .. 1908
Medical Record.. .. .. .. .. .. .. .. .. .. .. Vol. LXXIV 1908
Medical Review, The .. .. .. .. .. .. .. .. .. .. .. Vol. XI 1908
Medical Society's Transactions, The .. .. .. .. .. Vol. XXXI 1908
Münchener medizinische Wochenschrift .. .. .. .. Bd. II für 1908
New York Obstetrical Society, Transactions of the .. .. .. .. 1906–07
Pathological Society of London, Transactions of the (3) Vol. LVIII 1907
Practitioner, The .. .. .. .. .. .. .. .. .. .. .. [Vol. LXXXI] 1908
Progressive Medicine .. .. .. .. .. .. .. .. .. .. Vol. IV 1908
Quarterly Journal of Medicine .. .. .. .. .. .. Vol. I [1907]
Revue hebdomadaire de Laryngologie .. Tom. XXVIII, Vol. II 1908
Society for the Study of Disease in Children, Reports of the Vol. VIII, 1907–08; General Index, Vols. I–VIII 1908
Surgery, Gynecology and Obstetrics .. .. .. .. .. (5) Vol. IV 1907
Whitaker's Almanack .. .. .. .. .. .. .. .. .. .. .. .. .. .. 1909

**Contra-indications to the use of Milk in Disease,** by Dr. Michell Clarke.

**Infantile Diseases and Milk Diet,** by Dr. Bertram Rogers.

**The Heating of Milk as it affects the Nutrition and Health of the Infant,** by Dr. Fortescue-Brickdale.

For an abstract of these papers see previous pages of the *Journal.*

J. Lacy Firth,

J. A. Nixon, *Hon. Sec.*

---

### Local Medical Notes.

**University College, Bristol.**—The following examinations have recently been passed :—

M.D. Lond.—C. L. Morgan.†

M.S. Lond.—C. A. Moore,† W. R. Battye.

M.B., B.S. Lond.—C. Clarke, J. M. Hammond.† *Group II :* E. R. Holborow.

M.R.C.P. Lond.—J. W. Watson, I.M.S.†

F.R.C.S. Edin.—W. M. Bergin.†

Conjoint Examining Board.—*Chemistry :* R. I. Dacre. *Pharmacy :* G. H. Griffiths. *Anatomy and Physiology :* L. C. Watkins-Baker. *Medicine :* T. B. Dixon. *Surgery :* F. C. Nicholls, R. G. Vaughan.*

D.P.H. Irel.—*Honours :* Edmund J. F. Thomas.†

L.D.S. Eng.—*Dental Metallurgy :* F. J. Hayman, J. D. Melhuish, F. C. Willows, L. G. Kinnersley. *Mechanical Dentistry :* L. G. Kinnersley.

*Medical Entrance Scholarship :* C. C. Harrison, E. S. Sowerby (equal).

**The Radium Institute.**—Captain A. E. Hayward Pinch, late I.M.S., and formerly Superintendent of the Medical Graduates' College and Polyclinic, has been appointed Medical Director of this new institution. He was at one time a student and afterwards the Medical Tutor in the Bristol Medical School.

* Denotes qualification. † Former students.

**Bristol General Hospital.**—Charles Corfield, M.R.C.S., L.R.C.P. Lond., has been appointed Honorary Dental Anæsthetist, and Edward Emrys-Roberts, M.D., Ch.B. Vict., M.D., Ch.B. Liverp., has been appointed Pathologist.

**University College, Bristol.**—E. T. Glenny, M.B., B.S. Lond., has been appointed Demonstrator of Anatomy, and Edward Emrys-Roberts, M.D., Ch.B. Vict., M.D., Ch.B. Liverp., has been appointed Demonstrator of Pathology.

**Handel Cossham Hospital, Kingswood.**—James Young, M.D., C.M. Glasg., has been appointed Honorary Physician.

**Bristol University.**—At a meeting of the Committee of Bristol University, held on January 29th, it was announced that Lord Winterstoke had promised an additional £15,000 towards the University. This, with the £20,000 he had already given, makes Lord Winterstoke's contribution to the fund £35,000. A new movement in connection with Bristol University College and the proposed new University has recently been set on foot, a committee having been formed, with the Duchess of Beaufort as president, for establishing a hall of residence for women students. Contributions amounting to £775 have already been promised. For a permanent establishment some £10,000 will be needed, though for £2,000 temporary accommodation could be found.

**Bristol Dispensary.**—The annual meeting of the subscribers to the Bristol Dispensary was held on January 8th. The medical report showed that during the year 1908 10,957 patients had qeen treated, a considerable increase on previous years. The report added that during the year the Institution had lost the services of Dr. A. Carling, Dr. E. G. Hall, and Mr. J. B. Webb, after completing their full term of fifteen years' service, and of Dr. A. W. C. Richardson, who retired after two years' service from ill-health, and who has recently died. The Committee states that it is glad to place on record its appreciation of the good work done by these gentlemen.

**Bristol Eye Hospital.**—The annual meeting of the subscribers to the Bristol Eye Hospital was held on January 13th, under the presidency of the Lord Mayor. The medical report stated that during 1908 513 in-patients had been admitted, compared with 454 in 1907. The out-patients numbered 7,387, an increase of 592 on the previous year. The financial statement showed that the income falls short of the expenditure, and that the present deficit balance of the Institution was £641. In 1910 the committee hopes to celebrate the centenary of the Hospital, and although nothing definite has been decided upon, some suggested improvements in the building are under consideration.

**British Medical Library Association.**—At a meeting of those interested in Medical Libraries, held at Leeds on January 9th, it was decided unanimously to form an Association of Medical Libraries, and a provisional committee was appointed to draw up the constitution and rules. Professor W. Osler (Oxford) was invited to become the first president, and Dr. I. Walker Hall (Bristol) and Mr. Cuthbert E. A. Clayton (Librarian, Manchester Medical Society) were asked to undertake the duties of temporary secretaries. The following are some of the objects of the Association :—

1. Intercourse of those interested in medical library work, and the discussion of matters associated with the fostering and care of libraries.

2. Diffusion of information as to the branches of medical literature specially catered for at different centres, and as to the value of the various books and new periodicals which are issued from time to time.

3. The promotion of measures whereby a larger number of practitioners in each centre may be induced to utilise the library faculties of each district.

4. The consideration of matters connected with the present rapid increase of periodicals, publications of books, &c.

5. The opening up of better chances of advancement for library assistants. By such means there would be an inducement to parents to put their sons to such an occupation, and the librarians would be able to have better material for training.

6. The exchange of duplicate books and periodicals.

When the Association is in working order, it is hoped that a certain number of the libraries interested will join together to form a circle for the loan of their research and other literature.

**Royal United Hospital, Bath.**—The annual meeting of the subscribers of the Royal United Hospital, Bath, was held on January 27th. The medical report stated that during 1908 the in-patients admitted were 1,298, against 1,318 in 1907. The out-patients numbered 13,765, compared with 12,613 in the previous year. The financial statement showed an excess of expenditure over income of £1,020, which was due to a falling off in the amount of legacies. It was stated that a new building for the accommodation of the nurses was in course of erection.

**The late Dr. Emily Eberle.**—The honorary treasurer of "Dr. Emily Eberle Memorial Fund" has announced that the subscription list is now closed. £509 have been received, and this sum has been invested under a trust deed, and the income will be applied to the support of a bed in the Bristol Private

Hospital for Women and Children, Berkeley Square, Clifton, for the benefit of patients attending the Read Dispensary for Women, St. George's Road, Bristol.

**Territorial R.A.M.C.**—The 2nd Southern General Hospital, one of the twenty-three provided under the Territorial Force scheme, with its head-quarters at Bristol, has been in the process of construction during the past year, and in October last, having obtained 30 per cent. of its required strength, was recognised by the War Office.

Its function is to provide a base hospital of 500 beds, capable of considerable extension in or near Bristol, in the event of invasion. It is one of three base hospitals for the 7th District, which includes Warwickshire, Berkshire, Buckinghamshire, Worcestershire, Gloucestershire, and Oxfordshire Birmingham and Oxford being the head-quarters of the 1st and 3rd Southern General Hospitals. In Bristol the hospital will probably work in conjunction with the 3rd South Midland Field Ambulance and a Sanitary Corps, which as yet is not in being.

Unfortunately the 2nd Southern, as the other General Hospitals, has suffered from the slow and ponderous movements of the War Office and the County Associations; for after having been recognised for some four or five months, it has only just obtained temporary and very inadequate head-quarters, which it shares with the 3rd South Midland Field Ambulance, at the Drill Hall, Queen's Road, Clifton.

We hear that a movement which has been on foot for some considerable time to provide suitable quarters is slowly coming to a head, so it is possible that in the dim future suitable accommodation may be provided.

The permanent staff of the above consists of an Administrator, a Registrar, and a Quartermaster, who are as follows:—

J. Paul Bush, C.M.G., to be Administrator with the rank of Lieut.-Colonel.

H. L. Flemming to be Registrar with the rank of Major.

C. F. Walters, F.R.C.S., to be Quartermaster with the rank of Hon. Lieutenant.

The *à la suite* officers were reported in last number, Vol. XXVI, p. 380.

### Bristol Medico-Chirurgical Society.

### President.

J. MICHELL CLARKE, M.A., M.D. Cantab.

### President-Elect.

JAMES SWAIN, M.D., M.S. Lond.

### Committee.

*Ex-officio*:
- H. WALDO, M.D., C.M. Aberd. (Retiring President).
- R. SHINGLETON SMITH, M.D. Lond. (Editor of *Journal*).
- C. K. RUDGE, M.R.C.S. (Hon. Librarian).
- J. A. NIXON, B.A., M.B. Cantab. (Editorial Secretary).

*Elected*:

J. PAUL BUSH, C.M.G. }

JAMES TAYLOR, M.R.C.S. } Ex-Presidents.

B. M. H. ROGERS, M.A., M.D., B.Ch. Oxon.
P. WATSON WILLIAMS, M.D. Lond.
G. PARKER, M.A., M.D. Cantab.
I. WALKER HALL, M.D. Vict.
F. LACE, F.R.C.S.
J. LACY FIRTH, M.D., M.S. Lond.

### Honorary Secretary.

J. A. NIXON, B.A., M.B. Cantab.

Medical Practitioners wishing to join the Society are requested to communicate with the Hon. Secretary, Dr. NIXON, 18 West Mall, Clifton, Bristol. The Annual Subscription is 21/-.

---

*Extracts from Journal By-laws.*

4.—The *Journal* shall be delivered free to Subscribers and also to Members of the Society whose subscriptions are not in arrear.

6.—The Annual Subscription to the *Journal* for those who pay before the 1st of July shall be 5/-, post free, or 6/- if paid after that date.

---

Communications intended for insertion in the *Journal* should be sent to the Editor, Dr. SHINGLETON SMITH, Deepholm, Clifton Park, Clifton, Bristol.

Books for Review and Exchange Journals should be sent to the Assistant-Editor, *Bristol Medico-Chirurgical Journal*, Medical Department, University College, Bristol.

Communications referring to the delivery of the *Journal* should be sent to the Editorial Secretary, Dr. J. A. NIXON, 18 West Mall, Clifton, Bristol, to whom Subscriptions are to be paid *in advance*.

Cases for Binding Vols. I–XXVI are now ready, and may be obtained price 7*d.* each (postage 2*d.* extra), upon application to J. W. ARROWSMITH, Quay Street, Bristol; or the *Journal* will be bound, including Case, for 1/3 per volume.

### Bristol Medico-Chirurgical Society.

### PAST PRESIDENTS.

1874—75 FREDERICK BRITTAN, M.D., B.A. Dub.
1875—76. ROBERT WILLIAM COE.
1876—77. SAMUEL MARTYN, M.D. Ed.
1877—78. AUGUSTIN PRICHARD, M.D. Berlin.
1878—79. HENRY EDWARD FRIPP, M.D. Würzburg.
1879—80. WILLIAM MICHELL CLARKE.
1880—81. JOSEPH GRIFFITHS SWAYNE, M.D. Lond.
1881—82. EDWARD LONG FOX, M.D. Oxon.
1882—83. JAMES GEORGE DAVEY, M.D. St. And.
1883—84. WILLIAM JOHNSTONE FYFFE, M.D., B.A. Dub.
1884—85. GEORGE FORSTER BURDER, M.D. Aberd.
1885—86. WILLIAM HENRY SPENCER, M.D., M.A. Cantab.
1886—87. FRANCIS POOLE LANSDOWN.
1887—88. LEMUEL MATTHEWS GRIFFITHS.
1888—89. ROBERT SHINGLETON SMITH, M.D., B.Sc. Lond.
1889—90. NELSON CONGREVE DOBSON.
1890—91. SAMUEL HENRY SWAYNE.
1891—92. FRANCIS RICHARDSON CROSS, M.B. Lond.
1892—93. EDWARD MARKHAM SKERRITT, M.D., B.S., B.A. Lond.
1893—94. JAMES GREIG SMITH, M.B., C.M., M.A. Aberd., F.R.S.E.
1894—95. ALFRED JAMES HARRISON, M.B. Lond.
1895—96. ARTHUR WILLIAM PRICHARD.
1896—97. ALFRED EDWARD AUST LAWRENCE, M.D., C.M. Aberd.
1897—98. JOHN EDWARD SHAW, M.B., C.M. Ed.
1898—99. ROBERT ROXBURGH, M.B. Ed.
1899—00. WILLIAM HENRY HARSANT.
1900—01. DAVID SAMUEL DAVIES, M.D. Lond.
1901—02. BARCLAY JOSIAH BARON, M.B., C.M. Ed.
1902—03. GEORGE MUNRO SMITH.
1903—04. JAMES PAUL BUSH, C.M.G.
1904—05. REGINALD EAGER, M.D. Lond.
1905—06. JOHN DACRE.
1906—07. JAMES TAYLOR.
1907—08. HENRY WALDO, M.D., C.M. Aberd.

1909.

---

### LIST OF SUBSCRIBERS

TO THE

### Bristol Medico-Chirurgical Journal.

---

*Those marked * are Members of the Society.*

| | |
|---|---|
| ACKLAND, J. MCKNO ... ... ... ... ... ... | 1 Barnfield Crescent, Exeter. |
| *ACKLAND, W. R. ... ... ... ... ... ... ... ... | 5 Rodney Place, Clifton. |
| ADYE, W. J. A. ... ... ... ... ... ... ... ... .. | Church House Bradford, Wilts. |
| ALDOUS, GEORGE F.... ... ... ... ... ... ... | Charlton House, Mannamead, Plymouth |
| *ALEXANDER, D.A., M.B., Ch.B. Vict.... ... ... | 30 Berkeley Square, Clifton. |
| AMBROSE, J., M.B., C.M. Aberd. ... ... ... ... | 138 Fishponds Road, Eastville, Bristol. |
| AVELINE, H. T. S. ... ... ... ... ... ... ... ... | Somerset Asylum, Taunton. |

| | |
|---|---|
| *BALLANCE, H. STANLEY, M.D. Lond. ... ... | 4 Royal Terrace, Weston-super-Mare. |
| BARBER, M. C. ... ... ... ... ... ... ... ... | Staple Hill, Bristol. |
| BARETTI, T. G. L'ENARDI ... ... ... ... .. | 49 Royal York Crescent, Clifton. |
| *BARKER, G. H., M.B., B.Sc. Lond. ... ... ... | 124 Redland Road, Bristol |
| *BARON, BARCLAY J., M.B., C.M. Ed.... ... ... | 16 Whiteladies Road, Clifton. |
| BASSET, WALTER ... ... ... ... ... ... ... ... | 24 Stow Hill, Newport, Mon. |
| BATTEN, RAYNER W., M.D. Lond. ... ... ... | 1 Brunswick Square, Gloucester. |
| BATTEN, W. SMITH... ... ... ... ... ... ... | West Hill, Calne. |
| BAYER CO., THE ... ... ... ... ... ... ... ... | 19 St. Dunstan's Hill, London, E.C. |
| *BEAVER, R. A., M.D., Ch.B. Vict., M.B. Lond. | 15 The Avenue, Clifton. |
| *BEDDOE, JOHN, M.D. Ed., B.A. Lond., F.R.S. | The Chantry, Bradford-on-Avon. |
| BERNARD, C. ... ... ... ... ... ... ... ... ... | 2 Spencer Terrace, Fishponds. |

### LIST OF SUBSCRIBERS.

| | |
|---|---|
| *Berry, F. C., M.D., B.Ch., B.A. Dub. ... ... | Burnham, Somerset. |
| Berry, James, M.B., B.S. Lond. ... ... ... | 21 Wimpole Street, London, W. |
| Biamonti, Sebastiano ... ... ... ... ... ... ... | 27 Via Balbi, Genoa, Italy. |
| *Blacker, A. E.... ... ... ... ... ... ... ... ... | 20 Victoria Square, Clifton. |
| *Blacker, E., M.D. Durh. ... ... ... ... ... | 8 Meridian Road, Redland. |
| *Blackett, J. F., M.B., B.S. Lond. ... ... ... | Bristol Dispensary. |
| *Blackley, F. J., M.D. Dub. ... ... ... ... | 2 Knowle Road, Bristol. |
| *Blagg, Arthur F., M.D. Durh.... ... ... ... | 28 Caledonia Place, Clifton. |
| Board, J. H. ... ... ... ... ... ... ... ... ... | 8 Caledonia Place, Clifton. |
| Bodman, Hervey, M.D. Lond. ... ... ... ... | 3 Whiteladies Road, Clifton. |
| *Boucher, F. T. ... ... ... ... ... ... ... ... | Hazlemere, Cotham Road, Bristol. |
| *Bowker, G. E., M.D. Edin.... ... ... ... ... | 11 North Parade, Bath. |
| Boyce, James G. ... ... ... ... ... ... ... ... | The Chipping, Wotton-under-Edge. |
| Boyd, Geo. S. J. ... ... ... ... ... ... ... ... | 19 St. George's Square, Stamford, Lincs. |
| *Brasher, Charles W. J. ... ... ... ... ... | 128 Cotham Brow, Bristol. |
| *Brew, R. H. ... ... ... ... ... ... ... ... ... | Chew Magna. |
| *Bristowe, H. C., M.D. Lond. ... ... ... ... | Wrington, Somerset. |
| *Broom, H., M.B., C.M. Glas. ... ... ... ... | 47 City Road, Bristol. |
| Brown, G. Arthur ... ... ... ... ... ... ... | Tredegar. |
| Brown, William, M.D., C.M. Glasg. ... ... | Park View, Fishponds, Gloucestershire. |
| *Bullen, F. St. John ... ... ... ... ... ... ... | 12 Pembroke Road, Clifton. |
| *Bunbury, E. G. ... ... ... ... ... ... ... ... | 13 Dowry Square, Hotwells. |
| *Burd, E. Lycett, M.D., B.C. Cantab. ... ... | Shirehampton. |
| Burroughs, E. F. H. ... ... ... ... ... ... | 27 Burlington Road, Redland, Bristol. |
| *Bush, J. Paul, C.M.G. ... ... ... ... ... ... | Vyvyan House, Clifton Park, Clifton. |
| | |
| *Campbell-Horsfall, C.E., M.B., Ch.B. (Vict.) | Sisson Lodge, Newquay. |
| *Carter, T. M., M.D. Durh. ... ... ... ... | 28 Victoria Square, Clifton. |
| *Carwardine, Thomas, M.B., M.S. Lond. ... | 16 Victoria Square, Clifton. |
| *Cave, Edward J., M.D. Lond. ... ... ... ... | 20 Circus, Bath. |
| *Charles, J. R., M.A., M.D., B.C. Cantab. ... | 12 Victoria Square, Clifton. |
| *Chitty, H., M.S. Lond. ... ... ... ... ... | Bristol Royal Infirmary. |
| Chute, Henry M. ... ... ... ... ... ... ... ... | King William's Town, S. Africa. |
| *Clarke, John Michell, M.D., M.A. Cantab. | 28 Pembroke Road, Clifton. |
| Cobbledick, A. S., M.D., B.S. Lond. ... ... | 402 Brixton Road, London, S.W. |
| *Coleridge, Alfred, M.B., B.S. Lond. ... ... | 154 Coronation Road, Bristol. |
| *Collinson, T. A. ... ... ... ... ... ... ... ... | 62 Cranbrook Road, Redland, Bristol. |
| *Connellan, E. V. ... ... ... ... ... ... ... | Keynsham. |
| *Cook, Henri, M.D., C.M. Aberd. ... ... ... | 1 Dean Lane, Southville, Bristol. |
| *Coombs, Carey, M.D. Lond. ... ... ... ... | 74 St. Paul's Road, Clifton. |
| Cossham, W. R., M.D., C.M. Aberd.... ... ... | Cirencester. |
| Cotton, William, M.B., C.M., M.A. Ed. ... | 231 Gloucester Road, Bristol. |
| Coulson, Thos. E., M.B., C.M. Edin. ... ... | Brauncewell Manor, Sleaford, Lincs. |
| Coulter, R. J., M.B. Dub. ... ... ... ... ... | 11 Clytha Park Road, Newport, Mon. |
| Cridland, A. B. ... ... ... ... ... ... ... | 52 Waterloo Road, Wolverhampton. |
| *Cross, F. Richardson, M.B. Lond.... ... ... | Worcester House, Clifton. |
| *Crossman, F. W. ... ... ... ... ... ... ... ... | Hambrook, Gloucestershire. |
| *Crouch, C. Percival, M.B. Lond. ... ... ... | 1 Royal Terrace, Weston-super-Mare. |
| Culross, James, M.B., C.M., M.A. Glasg. ... | 66 Queen Street, Newton Abbot. |
| | |
| *Dacre, John ... ... ... ... ... ... ... ... ... | 14 Eaton Crescent, Clifton. |
| *Davies, D. S., M.D. Lond., D P.H. Cantab. ... | 60 Oakfield Road, Clifton. |
| *Davies, G. A. ... ... ... ... ... ... ... ... ... | 11 Alexandra Road, Clifton. |

### LIST OF SUBSCRIBERS.

| | |
|---|---|
| Dening, Edwin ... ... ... ... ... ... ... | Manor House, Stow-on-the-Wold. |
| Devine, H. ... ... ... ... ... ... ... ... ... | Long Grove, Epsom. |
| Dickie, James Steel, M.D., C.M. Aberd. ... | Boscombe Park, Bournemouth. |
| *Dobson, Nelson C. ... ... ... ... ... ... | 16 College Road, Clifton. |
| *Dowling, E. A. G. ... ... ... ... ... ... | 5 Rodney Place, Clifton. |
| Dunbar, Eliza L. Walker, M.D. Zürich ... | 9 Oakfield Road, Clifton. |
| *Dunkley, E. V., M.B., B.S. Lond. ... ... ... | |
| Dymoke, F. ... ... ... ... ... ... ... ... | 21 Cotham Road, Bristol. |

| | |
|---|---|
| Eadon, W. F. Bailey ... ... ... ... ... ... | Hambrook, Gloucestershire. |
| *Eager, Reginald, M.D. Lond. ... ... ... ... | Northwoods, Winterbourne. |
| *Edgeworth, F. H., M.B., B.C., B.A. Cantab., D.Sc. Lond. ... ... ... ... ... ... ... ... | 35 Pembroke Road, Clifton. |
| Edwards, E. S. ... ... ... ... ... ... ... | Empingham, Stamford. |
| Elder, William ... ... ... ... ... ... ... | 4 Trelawney Road, Bristol. |
| Elliot Bros. Ltd. ... ... ... ... ... ... | *Australian Pharmaceutical Notes & News.* O'Connell Street, Sydney, N.S.W. |
| *Elliott, Christopher, M.D., B.A. Dub. ... | 3 Beaufort Road, Clifton. |
| Elliott, F. Percy, M.B., C.M. Aberd. ... ... | 113 Grove Road, Walthamstow, London. N.E. |
| *Elliott, W. H. A., M.B., B.S. Lond. ... ... | Rockfort, Arley Hill, Bristol. |
| *Emrys-Roberts, E., M.D. Liverpl. ... ... | 5 Buckingham Place, Clifton. |
| Evans, T. G. C. ... ... ... ... ... ... ... | Budleigh Salterton, Devon. |
| *Every-Clayton, L. E. V., M.D. Lond. ... ... | Rosslyn, Clevedon, Somerset. |

| | |
|---|---|
| *Fells, A., M.B., C.M. Edin. ... ... ... ... | 107 Ashley Road, Bristol. |
| *Ferguson, R. S., B.A. Durh., M.B. C.M. Edin. | Elm Grove, Calne, Wilts. |
| *Firth, J. L., M.D., M.S. Lond. ... ... ... ... | 8 Victoria Square, Clifton. |
| *Fleck, D., M.B., B.Ch., R.U.I. ... ... ... ... | Brentry Inebriate Home, Westbury-on-Trym. |
| *Flemming, A. L. ... ... ... ... ... ... ... | 34 Alma Road, Clifton. |
| *Flemming, C. E. S. ... ... ... ... ... ... | Manvers House, Bradford-on-Avon. |
| *Fletcher, J., M.D., C.M., D.P.H. Aberd. ... | Ham Green, Somerset. |
| Flood, P. ... ... ... ... ... ... ... ... ... | 318 Stapleton Road, Bristol. |
| *Fortescue-Brickdale, J. M., M.A., M.D., B.Ch. Oxon. ... ... ... ... ... ... ... | 52 Pembroke Road, Clifton. |
| Forty, D. H. ... ... ... ... ... ... ... ... | Wotton-under-Edge. |
| Foss, E. V. ... ... ... ... ... ... ... ... | Clouds Hill House, St. George, Bristol. |
| Fox, F. F. ... ... ... ... ... ... ... ... ... | Yate House, Yate. |
| *Fraser, Forbes ... ... ... ... ... ... ... | 2 The Circus, Bath. |
| Freeman, J., M.D. Brux. ... ... ... ... ... | Glencairn Villa, Queen's Road, Clifton. |

| | |
|---|---|
| Garland, E. B., M.B., C.M. Edin. ... ... ... | 114*a* Hampton Road, Redland. |
| Genge, T. T. ... ... ... ... ... ... ... ... | 21 Whiteladies Road, Bristol. |
| *Gerrish, D. S. ... ... ... ... ... ... ... ... | 322 Church Road, St. George, Bristol. |
| *Gibbs, A. N. Godby ... ... ... ... ... ... | 52 Whiteladies Road, Clifton. |
| *Glenny, E. T., M.B., B.S. Lond. ... ... ... | 61 Effingham Road, Bristol. |

### LIST OF SUBSCRIBERS.

Goss, T. Biddulph... ... ... ... ... ... ... ... 1 The Circus, Bath.
*Goulden, C. B., M.A., M.B., M.C. Cantab. ...
*Gratte, C. Brooke ... ... ... ... ... ... 19 Gold Tops, Newport, Mon.
*Green, T. A., M.D., C.M. Edin. ... ... ... 33*a* Whiteladies Road, Clifton.
Green-Armytage, Lt. V. B., R.A.M.C. ... c/o King & Co., Bombay.
*Greer, W. J. ... ... ... ... ... ... ... ... 19 Gold Tops, Newport, Mon.
*Grey, Harry, M.D., C.M.Edin. ... ... ... 492 Fishponds Road, Bristol.
Griffiths, Cornelius A. ... ... ... ... ... 6 Windsor Place, Cardiff.
*Griffiths, J. S. ... ... ... ... ... ... ... 20 Redland Park, Bristol.
*Griffiths, L. M. ... ... ... ... ... ... ... 11 Pembroke Road, Clifton.
*Groves, E. W. H., M.B., B.Sc. Lond. ... ... 16 Richmond Hill, Clifton.

Hadwen, W. R., M.D. St. And. ... ... ... ... 35 Brunswick Square, Gloucester.

*Hailes, Clements D. G., M.D., C.M. Ed. ... 27 Alma Road, Clifton.

*Haines, A. H. ... ... ... ... ... ... ... ... 12D Cotham Road, Bristol.

*Hall, E. G., M.B. Lond. ... ... ... ... ... 53 Hampton Road, Redland, Bristol.

*Hall, I. Walker, M.D., Ch.B.Vict. ... ... 9 Royal Park, Clifton.

Handfield-Jones, Montagu, M.D. Lond. ... 35 Cavendish Square, London, W.

*Harris, H. Elwin, B.A., M.B. Cantab. ... ... 13 Lansdown Place, Clifton.

*Harrison, A. J., M.B. Lond. ... ... ... ... Guthrie Road, Clifton.

*Harsant, W. H. ... ... ... ... ... ... ... Tower House, Clifton.

Hartnell, E. B. ... ... ... ... ... ... ... ... Thornbury, R.S.O., Glos.

*Harvey, Alfred, M.B. Lond. ... ... ... ... Westbury-on-Trym.

*Hawes, C. S. ... ... ... ... ... ... ... ... Nordrach-upon-Mendip, Blagdon.

*Hawes, I. H., M.B., B.S. Durh. ... ... ... Wick, Glos.

Hayman, Alfred G. ... ... ... ... ... ... ... Hapsford House, near Frome.

*Hayman, C. A., M.D. St. And. ... ... ... ... Kingston Villa, Richmond Hill, Clifton

*Herapath, C. E. K., M.B. Lond. ... ... ... 12 Brunswick Square, Bristol.

*Herapath, C. K. C. ... ... ... ... ... ... Cotham Park, Bristol.

Hill, Hedley ... ... ... ... ... ... ... ... ... 1 Whiteladies Road, Clifton.

*Hill, Walter J. ... ... ... ... ... ... ... ... The Brow, Clevedon.

Hodges, W. ... ... ... ... ... ... ... ... ... 38 Park Road, Gloucester.

Hospital, Bristol General (*Sec.*, W. Thwaites).

Hospital, Taunton and Somerset (*House-Surgeon*, W. Drake).

Huggard, W. R., M.D., M.Ch., M.A. Roy. Univ. ... ... ... ... ... ... ... ... ... Davos-Platz, Switzerland.

Imlay, G. A., M.B., C.M. Aberd. ... ... ... 1 Cotham Park, Bristol.
Infirmary, Bristol Royal (*Secretary*, W. E. Budgett).
Ingle, C. Durban ... ... ... ... ... ... ... Somerton, Somerset.
*Irwin, S., M.B., B.Ch., B.A.O., R.U.I. ... ... Olveston, Glos.

Jamison, A., M.B., B.Ch., M.A. R.U.I. ... ... Woodstock Road, Belfast.
Jones, Evan ... ... ... ... ... ... ... ... ... Ty-mawr, Aberdare.
*Jones, E. J. Trevor, M.D. Brux. ... ... ... Aberdare, Glamorganshire.
*Jones, J. Ellington ... ... ... ... ... ... 16 Richmond Park Road, Clifton.

### LIST OF SUBSCRIBERS.

| | |
|---|---|
| JONES, H. MACNAUGHTON, M.D., M.Ch. R.U.I. | 131 Harley Street, London, W. |
| *JONES, R. LLEWELLYN, M.B. Lond. ... ... ... | 40 Gay Street, Bath. |
| JOSEPH, A. HILL, M.D. Lond. ... ... ... ... | Bexhill-on-Sea. |
| *KEIR, I. C., M.B., Ch.B. Ed. ... ... ... ... | The Limes, Melksham. |
| *KEMM, F. ST. J.... ... ... ... ... ... ... ... | Worle. |
| *KYLE, H. G., M.B., B.Ch. Oxon. ... ... ... | 31 Westbury Road, Westbury-on-Trym. |
| *LACE, F. ... ... ... ... ... ... ... ... ... | 1 Paragon, Bath. |
| *LANSDOWN, R. G. P., M.B., B.S. Dur. ... ... | 39 Oakfield Road, Clifton. |
| *LAVINGTON, C. C., M.B., B.S. Dur. ... ... | 1 Burlington Road, Redland. |
| LENDON, A. A., M.D. Lond. ... ... ... ... | North Terrace, Adelaide, S. Australia. |
| LE SOUDIER, H. ... ... ... ... ... ... ... ... | 174 et 176 Boulevard St. Germain, Paris. |
| *LEES, E. LEONARD, M.D., C.M. Ed. ... ... ... | 1 Chesterfield Place, Clifton. |
| LEIGH, W. W. ... ... ... ... ... ... ... ... | Treharris, Glamorganshire. |
| *LEONARD, R. C., M.D. Ed. ... ... ... ... ... | 134 Coronation Road, Bristol. |
| *LUCAS, J. J. S., M.D. Lond. ... ... ... ... ... | 186 Wells Road, Totterdown. |
| LUCY, REGINALD H. ... ... ... ... ... ... | 9 The Crescent, Plymouth. |
| *McCREA, P. W., M.D., B.Ch., B.A.O., B.A. Dub. ... ... ... ... ... ... ... ... ... ... | 19 Portland Square, Bristol. |
| MAC DONALD, P. W., M.D., C.M. Aberd. ... ... | Herrison, Dorchester. |
| MACKINNON, CHARLES, M.B., C.M., M.A. Glasg. | Davaar, Cirencester. |
| MACLEAN, EWEN J., M.D., C.M. Ed.... ... ... | 12 Park Place, Cardiff. |
| MACLEAN, J. CAMPBELL, M.B., C.M. Ed. ... ... | Swindon House, Swindon, Wiltshire. |
| *MACLEOD, D., M.B., B.C. Ed. ... ... ... ... | 84 Redland Road, Bristol. |
| MACREADY, J. ... ... ... ... ... ... ... ... ... | 42 Devonshire Street, Portland Place, London, W. |
| *MACWATTERS, J. C. ... ... ... ... ... ... ... | Almondsbury. |
| MARSH, O. E. BULWER ... ... ... ... ... ... | Parkdale, Newport, Mon. |
| MARSHALL, ARTHUR L., M.B., B.A. Cantab ... | 206 Upper Clapton Road, London, N.E. |
| *MARTIN, E. F., M.B. Ed. ... ... ... ... ... ... | 7 Royal Terrace, Weston-super-Mare. |
| *MARTIN, R C. ... ... ... ... ... ... ... ... ... | 3 Clifton Vale, Clifton. |
| *MARTYN, G. J. KING, M D., B.C., B.A. Cantab. ... ... ... ... ... ... ... ... ... | 12 Gay Street, Bath. |
| MASON, S. B. ... ... ... ... ... ... ... ... | 12 Park Terrace, Pontypool. |
| MASON, WILLIAM ... ... ... ... ... ... ... ... | Sedgemoor Cottage, St. Austell, Cornwall. |
| MATTHEWS, CHARLES E., M.D. Dur. ... ... | St. Aubyn's Park, Tiverton. |
| MEREDITH, JOHN, M.D. Ed. ... ... ... ... ... | Wellington, Somerset. |
| *MOLE, HAROLD F. ... ... ... ... ... ... ... ... | 19 Mortimer Road, Clifton, Bristol. |
| MONCKTON, WILLIAM ... ... ... ... ... ... | Portishead. |

### LIST OF SUBSCRIBERS.

| | |
|---|---|
| *Moore, C. A., M.B., B.S. Lond. ... ... ... ... ... | General Hospital, Bristol. |
| *Moore, L. A. ... ... ... ... ... ... ... ... ... ... | 45 Ashley Road, Bristol. |
| Moores, De la Hey ... ... ... ... ... .. ... | 6 West Mall, Clifton. |
| *Morgan, T. Whitworth S. ... ... ... ... | The Laurels, Timsbury, Bath. |
| *Morton, C. A. ... ... ... ... ... ... ... ... ... | 14 Vyvyan Terrace, Clifton. |
| *Morton, W. B., M.D. Lond. ... ... ... ... | Brislington House, Brislington. |
| Munden, Charles ... ... ... ... ... ... ... ... | Ilminster. |
| *Munro, J. M. H., D.Sc. Lond. ... ... ... ... | 12 Grosvenor Place, Bath. |
| *Muthu, Chowry, M.D. Brux. ... ... ... ... | Mendip Hills Sanatorium, Wells. |
| *Myles, G T. ... ... ... ... ... ... ... ... ... | 147 Whiteladies Road, Bristol. |

| | |
|---|---|
| *Neild, Newman, M.B., B.Ch. Vict. ... ... ... | 9 Richmond Hill, Clifton. |
| *Newman, Charles ... ... ... ... ... ... ... ... | 156 Cheltenham Road, Bristol. |
| *Newman, Herbert, M.B., B.S. Dur. ... .... | 102 Coronation Road, Bristol. |
| *Newnham, W. H. C., M.B., M.A. Cantab. ... | 3 Lansdown Place, Victoria Square, Clifton. |
| Nicholson, T. D., M.D. Ed. ... ... ... ... | 2 Whiteladies Road, Clifton. |
| *Nixon, J. A., B.A., M.B., B.C. Cantab. ... ... | 18 West Mall, Clifton. |
| Norman, J. H. ... ... ... ... ... ... ... ... | Goodleigh, Winkleigh, Devonshire. |

| | |
|---|---|
| Odell, William, M.D. Durh. ... ... ... ... | Ferndale, Torquay. |
| *Ogilvy, Alexander, M.D., B.Ch., B.A. Dub. | Leny, Clifton Park, Clifton. |
| Openshaw, E. H. ... ... ... ... ... ... ... ... | Cheddar. |
| *Ormerod, H. Lawrence, M.B., B.Ch. R.U.I. | 2 Henleaze Road, Westbury Park. |

| | |
|---|---|
| Page, G. Shepley ... ... ... ... ... ... ... ... | 78 Old Market Street, Bristol. |
| Papillon, J. W. ... ... ... ... ... ... ... ... | Brent Knoll, Somerset. |
| *Parker, George, M.D., M.A. Cantab. ... ... | 14 Pembroke Road, Clifton. |
| Parkinson, Lt. G. S., R.A.M.C. ... ... ... | Military Hospital, Belfast. |
| *Parsons, H. F. ... ... ... ... ... ... ... ... | The Heath, Stoke Bishop. |
| Parsons, J. H., M.B., B.S., D.Sc. Lond. ... ... | 27 Wimpole Street, London, W. |
| Paterson, Donald Rose, M.D., C.M. Ed. ... | 18 Windsor Place, Cardiff. |
| Peake, Arthur W. ... ... ... ... ... ... ... | 34 Gloucester Road, Bishopston, Bristol. |
| Peake, G. Arthur ... ... ... ... ... ... ... | Rodney Place, Cheltenham. |
| Pearse, E. M. ... ... ... ... ... ... ... ... | 2 Varley Terrace, Liskeard. |
| Perdrau, J. A., M.B. Lond. ... ... ... ... | 99 Gower Street, London, W.C. |
| Phillips, C. M., M.D. Brux. ... ... ... ... | Ravenslea, Kensington Hill, Brislington |
| Phillips, Edward Picton ... ... ... ... ... | High Street, Haverfordwest. |
| *Pickering, C. F. ... ... ... ... ... ... ... | 13 Berkeley Square, Bristol. |
| *Pinniger, W. J. H., M.D., B.S. Lond. ... ... | 9 Beaconsfield Road, Clifton. |
| Powell, J. J., M.D. Lond. ... ... ... ... ... | 2 Gloucester Road, Bishopston, Bristol. |
| Powell, William, M.B. Lond. ... ... ... ... | Hill Garden, Torquay. |
| Price, D. T., M.B. Lond. ... ... ... ... ... | Castle Cary, Somerset. |

### LIST OF SUBSCRIBERS.

*Prichard, Arthur W. ... ... ... ... ... ... 6 Rodney Place, Clifton.
*Prowse, Arthur B., M.D. Lond. ... ... ... 5 Lansdown Place, Clifton.
Prowse, W. B.... ... ... ... ... ... ... ... 11 St. George's Place, Brighton.

Randolph, Charles ... ... ... ... ... ... St. Michael's, Milverton, Somerset.
*Rattray, J. M., M.D., C.M., M.A. Aberd. ... Frome.
*Rayner, D. C. ... ... ... ... ... ... ... ... 9 Lansdown Place, Clifton, Bristol.
Redwood, T. Hall, M.D., C.M., M.A.Dur. ... The Lawn, Rhymney, Monmouthshire.
Rendall, John ... ... ... ... ... ... ... Forestside, Lymington.
*Ridge, Percy B., M.B., B.Ch., R.U.I. ... ... Children's Hospital, Bristol.
Ritchie. Thomas P. ... ... ... ... ... ... 3 Redcliff Parade West, Bristol.
*Rogers, B. M. H., M.D., B.Ch., B.A. Oxon. ... 1 Victoria Square, Clifton.
*Rolfe, R., B.A., M.B., B.C. Camb. ... ... ... Park House, Avonmouth.
*Rose, F. H. ... ... ... ... ... ... ... ... 13 Westfield Park, Clifton.
*Rossiter, George F., M.B. Lond. ... ... Cairo Lodge, Weston-super-Mare.
*Roué, W. Barrett, M.D., M.S. Dur. ... ... 2 Buckingham Place, Clifton.
*Roxburgh, Robert, M.B. Ed. ... ... ... 1 Victoria Buildings, Weston-super-Mare
*Rudge, C. King ... ... ... ... ... ... ... 145 Whiteladies Road, Bristol.
*Rudge, F. H. ... ... ... ... ... ... ... ... 145 Whiteladies Road, Bristol.
Ruffer, M. Armand, M.D., B.S., M.B. Oxon Ramleh, Alexandria, Egypt.

Scott, Baty- ... ... ... ... ... ... ... ... 5 Haverstock Road, Knowle, Bristol.
*Schölberg, H. A., M.B., B.S. Lond. ... ... University College, Cardiff.
Seward, W. J., M.B. Lond. ... ... ... ... Colney Hatch Asylum, New Southgate, London, N.
*Shaw, J. E., M.B., C.M. Ed. ... ... ... ... 23 Caledonia Place, Clifton.
Sheppard, A. L., M.B., B.S.Dur. ... ... ... Selwood, Frome.
*Sheppard, E. J. .. ... ... ... .. ... ... 46 St. Paul's Road, Clifton.
*Shoolbred, W. A. ... ... ... ... ... ... ... St. Ann's, Chepstow.
*Short, A. R., M.D., B.S., B.Sc. Lond. ... ... 2 Westbourne Place, Queen's Road, Clifton.
*Short, L. J., M.B., B.Sc. Lond. ... ... ... 21 Tyndall Avenue, Clifton.
Skelton, Henry, M.D. St. And. ... ... ... Downend, Gloucestershire.
Smith, F. Shingleton, B.A., B.C.Cantab. ... Grindlay & Co., Bombay.
*Smith, G. Cockburn, M.D. Brux. ... ... ... 14 South Road, Newton Abbot.
*Smith, G. Munro ... ... ... ... ... ... ... 18 Apsley Road, Clifton.
*Smith, L. Shingleton, B.A., M.B. Cantab.... Wotton House, Brecon.
Smith, Rev. Reginald, M.A. Cantab. ... ... The Vicarage, Walpole St. Andrew, Norfolk.
*Smith, R. Shingleton, M.D., B.Sc. Lond. ... Clifton Park, Clifton.
Smith, W., M.B. Lond.... ... ... ... ... ... 4 Ardgowan Square, Greenock, N.B.
*Smith, W. Alexander, M.B., M.A. Oxon. ... 14 Durdham Park, Redland, Bristol.
Society, Cardiff Medical (*Hon. Lib.*, Queen Street, Cardiff).

### LIST OF SUBSCRIBERS.

| | |
|---|---|
| Society, Manchester Medical | Medical Society's Library, The Owens College, Manchester. |
| Society, Plymouth Medical (*Hon. Lib.*, J. Elliott Square). | |
| *Stack, E. H. E., B.A., M.B., B.C. Cantab. | Arvalee, Clifton Down Road, Clifton. |
| Staple, J. D. | Clevedon Villa, Cheltenham Road, Bristol. |
| Statham, R. Whiteside | Cheddar, Somersetshire. |
| *Steele, Charles, M.D. Dur. | 17 Richmond Hill, Clifton. |
| Stevens, W. H. | 26 Zetland Road, Bristol. |
| Stock, P. G. | Box 1049, Johannesburg, South Africa. |
| *Stock, W. S. V., M.B., B.S. Lond. | Montrose House, Queen's Road, Clifton. |
| Stoddart, F. Wallis | Southey House, College Green, Bristol. |
| *Swain, James, M.D., M.S. Lond. | 4 Victoria Square, Clifton. |
| *Swayne, W. Carless, M.D. Lond. | St. Paul's Road, Clifton. |
| *Symes, J. O., M.D. Lond. | 71 Pembroke Road, Clifton. |
| Symonds, H. P. | 35 Beaumont Street, Oxford. |

| | |
|---|---|
| Taylor, A. L. | 22 Downfield Road, Clifton. |
| Taylor, C. Bell, M.D. Edin. | 9 Park Row, Nottingham. |
| Taylor, C. J. | 43 Duke Street, Chelmsford. |
| *Taylor, James | 36 Alma Road, Clifton. |
| *Temple, G. H., M.B., C.M. Ed. | Weston-super-Mare. |
| Thin, James | 54 & 55 South Bridge, Edinburgh. |
| Thomas, A. Garrod, M.D., C.M. Ed. | Clytha Park, Newport, Mon. |
| *Thomas, J. D., M.B. Cantab. | Northwoods, Winterborne, |
| Thomas, J. Lynn, C.B. | 21 Windsor Place, Cardiff. |
| *Thomas, J. M. M. | 5 Colston Parade, Redcliffe, Bristol. |
| *Thomas, J. Tubb | The Halve House, Trowbridge. |
| Thomson, Eustace B., M.D., C.M. Glasg. | Albany Place, Plymouth. |
| Tivy, W. J. | 5 Victoria Square, Clifton. |
| Tosswill, Leonard R., M.B., B.C. Camb. | Devon County Council Offices, 14 Bedford Circus, Exeter. |
| Tyley, R. P., M.D. St. And. | Wedmore. |

| | |
|---|---|
| Vachell, Herbert R., M.D., C.M. Aberd. | 18 Newport Road, Cardiff. |
| *Vickery, W. H. | 1 Trewartha Park, Weston-super-Mare. |

| | |
|---|---|
| *Waldo, Henry, M.D., C.M. Aberd. | 19 Pembroke Road, Clifton. |
| *Walker, Cyril H., M.B., B.A. Cantab. | 8 Oakfield Road, Clifton. |
| Wallace, Rev. Canon, M.A. Oxon. | 3 Gloucester Row, Clifton. |
| *Wallace, John | Lauriston, 15 Knightstone Road, Weston-super-Mare. |
| Wallace, J. T., M.B., B.Ch., B.A. R.U.I. | 48 Coronation Road, Bedminster. |
| *Walters, C. F. | 19 Whatley Road, Clifton. |
| *Waterhouse, R., M.D. Lond. | 32 St, James's Square, Bath. |
| Webber, William W. | Crewkerne. |
| *Westcott, Staff-Surgeon W. G. | H.M.S. *Adventure*, Harwich. |
| *Whicher, A. H. | 115 Queen's Road, Clifton |

### LIST OF SUBSCRIBERS.

| | |
|---|---|
| WHITE, C. R. ... ... ... ... ... ... ... ... | Nixon Villa, Merthyr Vale, Glamorgan. |
| WHITE, J. W. ... ... ... ... ... ... ... ... | Orchard House, Nailsea. |
| *WHITE, PERCY W. ... ... ... ... ... ... ... | College Green, Bristol. |
| WIGAN, C. A., M.D. Dur. ... ... ... ... ... | Portishead. |
| WIGMORE, J., M.D. Roy. Univ. I. ... ... ... | 20 Green Park, Bath. |
| WILCOX, HENRY, M.B. Aberd. ... ... ... ... | 10 Willington Road, Eastbourne. |
| WILLCOX, R. LEWIS ... ... ... ... ... ... ... | Warminster. |
| WILLETT, GEORGE G. D. ... ... ... ... ... ... | Keynsham, Somerset. |
| *WILLIAMS, E. C., M.B., B.C., B.A. Cantab. ... | 159 Whiteladies Road, Clifton. |
| WILLIAMS, H. EGERTON ... ... ... ... ... ... | The Mount, Newport, Mon. |
| *WILLIAMS, P. WATSON, M.D. Lond.... ... ... | 4 Clifton Park, Clifton. |
| *WILLIAMS, W. ROGER ... ... ... ... ... ... | Morven, Walton-by-Clevedon. |
| WILLIS, W. M.... ... ... ... ... ... ... ... | 7 Regent Street, Nottingham. |
| *WILLS, W. K., M.B., B.C. Camb. ... ... ... | 19 Whiteladies Road, Clifton. |
| *WINDSOR-AUBREY, H. W. ... ... ... ... ... | Coronation Road, Bristol. |
| *WOOD, W. V. ... ... ... ... ... ... ... ... | Glencairn, Wrington. |
| WOOLLCOMBE, WALTER L. ... ... ... ... ... | 16 The Crescent, Plymouth. |
| YOUNG, J., M.D. Glas. ... ... ... ... ... ... | 14 Chantry Road, Clifton. |

### Bristol Medico-Chirurgical Journal.

**TERMS FOR ADVERTISEMENTS.**

| | £ | s. | d. |
|---|---|---|---|
| Page ... ... ... ... ... ... ... ... ... ... ... ... ... | 1 | 1 | 0 |
| Half Page ... ... ... ... ... ... ... ... ... ... ... | 0 | 12 | 6 |
| Quarter Page ... ... ... ... ... ... ... ... ... ... ... | 0 | 7 | 6 |

Insets (2 or 4 pages), 20/-
Terms for special positions upon application.

*Orders to be sent to the Editorial Secretary,*
Dr. J. A. NIXON, 18 West Mall, CLIFTON.
*or the Publisher,* J. W. ARROWSMITH, Printer, 11 Quay Street, BRISTOL.

THE TRANSACTIONS

OF THE

OPHTHALMOLOGICAL SOCIETY

